# Host specificity of *Enterocytozoon bieneusi* genotypes in Bactrian camels (*Camelus bactrianus*) in China

**DOI:** 10.1186/s13071-018-2793-9

**Published:** 2018-04-02

**Authors:** Meng Qi, Junqiang Li, Aiyun Zhao, Zhaohui Cui, Zilin Wei, Bo Jing, Longxian Zhang

**Affiliations:** 1 College of Animal Science, Tarim University, Alar, Xinjiang, 843300 China; 2 College of Animal Science and Veterinary Medicine, Henan Agricultural University, Zhengzhou, 450002 China

**Keywords:** *Enterocytozoon bieneusi*, Bactrian camel, Genotype, Host specificity

## Abstract

**Background:**

*Enterocytozoon bieneusi* is an obligate, intracellular fungus and is commonly reported in humans and animals. To date, there have been no reports of *E. bieneusi* infections in Bactrian camels (*Camelus bactrianus*). The present study was conducted to understand the occurrence and molecular characteristics of *E. bieneusi* in Bactrian camels in China.

**Results:**

Of 407 individual Bactrian camel fecal specimens, 30.0% (122) were *E. bieneusi*-positive by nested polymerase chain reaction (PCR) based on internal transcriber spacer (ITS) sequence analysis. A total of 14 distinct *E. bieneusi* ITS genotypes were obtained: eight known genotypes (genotype EbpC, EbpA, Henan-IV, BEB6, CM8, CHG16, O and WL17), and six novel genotypes (named CAM1 to CAM6). Genotype CAM1 (59.0%, 72/122) was the most predominant genotype in Bactrian camels in Xinjiang, and genotype EbpC (18.9%, 23/122) was the second-most predominant genotype. Phylogenetic analysis revealed that six known genotypes (EbpC, EbpA, WL17, Henan-IV, CM8 and O) and three novel genotypes (CAM3, CAM5 and CAM6) fell into the human-pathogenic group 1. Two known genotypes (CHG16 and BEB6) fell into the cattle host-specific group 2. The novel genotypes CAM1, CAM 2 and CAM4 cluster into group 8.

**Conclusions:**

To our knowledge, this is the first report of *E. bieneusi* in Bactrian camels. The host-specific genotype CAM1 was the predominant genotype, which plays a negligible role in the zoonotic transmission of *E. bieneusi*. However, the second-most predominant genotype, EbpC, has greater zoonotic potential.

## Background

Microsporidia are a diverse group of emerging obligate intracellular eukaryotic fungi and there are approximately 1300 microsporidian species in 160 genera [1]. To date, there are at least 14 microsporidian species reported to be infectious to humans [2]. *Enterocytozoon bieneusi* is the most frequently detected species in humans [3], as well as in domestic animals and wildlife [4], and even in environmental water samples [5].

More than 200 *E. bieneusi* genotypes have been identified in humans and animals by polymerase chain reaction (PCR) based on ribosomal internal transcribed spacer (ITS) gene sequence analysis [2, 6]. Molecular phylogenetic analysis has shown that all *E. bieneusi* ITS genotypes are clustered into nine large groups, including the potentially zoonotic group 1, and some host-specific groups (Group 2 to Group 9) [7].

The Bactrian camel (*Camelus bactrianus*) was the major means of transportation on the ancient Silk Road. Today, the population of Bactrian camels in China has been estimated at 242,000, most of which are domesticated in desert and semi-desert areas of northwestern China and play an important role in the livelihood of pastoralists through providing milk and meat [8]. There are some reports of intestinal pathogen infections in camels and Bactrian camels in the Middle East countries and China, such as *Eimeria* spp. and *Cryptosporidium* spp. [9, 10]. However, *E. bieneusi* infection has not been previously reported in Bactrian camels.

This study was undertaken to better understand the prevalence of *E. bieneusi* in Bactrian camels and assess the host specificity of *E. bieneusi* infections in Bactrian camels in China.

## Methods

### Specimen collection

A total of 407 individual fresh fecal specimens from Bactrian camels were collected from 18 different grazing Bactrian camel groups in 11 collection sites of Xinjiang Uygur Autonomous Region (hereinafter referred to as Xinjiang) of northwestern China. Only one specimen was collected per animal. These specimens were collected during August and September of 2013 and from July 2016 to July 2017 (Table 1). The grazing Bactrian camel groups were kept outdoors and shared pastures with cattle, sheep, goats and wild animals, and each group had approximately 30–300 animals.

**Table 1:** The infection status of *E. bieneusi* and genotypes in Bactrian camels in Xinjiang, China

Collection site	Collection time	No. of specimens	No. infected (%)	Genotypes (no.)
Urumqi-1	August 2013	13	11 (84.6)	CAM1 (11)
Urumqi-2	August 2013	41	11 (26.8)	EbpC (5), CAM1 (3), EbpA (2), CAM4 (1)
Urumqi-3	August 2013	46	15 (32.6)	EbpC (7), CAM1 (3), CAM4 (2), BEB6 (1), EbpA (1), CAM2 (1)
Urumqi-4	August 2013	13	3 (23.1)	EbpC (1), CAM5 (1), O (1)
Urumqi-5	August 2013	17	9 (52.9)	CAM1 (4), EbpC (2), EbpA (2), CAM4 (1)
Urumqi-6	August 2013	13	4 (30.8)	CAM1 (1), EbpC (1), CAM3 (1), CAM6 (1)
Urumqi-7	August 2013	25	7 (28.0)	CAM1 (6), CHG16 (1)
Urumqi-8	August 2013	14	5 (35.7)	CAM1 (3), EbpC (2)
Fukang	August 2013	41	19 (46.3)	CAM1 (11), EbpC (3), CAM2 (2), Henan-IV (1), CM8 (1), WL17 (1)
Altai	September 2013	11	3 (27.3)	EbpC (2), CAM1 (1)
Bachu	July 2016	17	0	
Wensu	August 2016	7	0	
Qitai	September 2016	18	3 (16.7)	CAM1 (2), CAM4 (1)
Pishan	October 2016	17	3 (17.6)	CAM1 (3)
Barkol	December 2016	32	0	
Qinghe	February 2017	10	2 (20.0)	CAM1 (1), CAM2 (1)
Shihezi	July 2017	60	27 (45.0)	CAM1 (23), CAM2 (4)
Chabucha'er Xibo Zizhixian	July 2017	12	0	
Total		407	122 (30.0)	CAM1 (72), EbpC (23), CAM2 (8), EbpA (5), CAM4 (5), Henan-IV (1), BEB6(1), CM8 (1), CHG16 (1), O (1), WL17 (1), CAM3 (1), CAM5 (1), CAM6 (1)

After animal defecation, about 50–100 g of each fresh specimen was collected immediately from the ground using sterile gloves. Each specimen was collected in a plastic container and marked with the specimen number and site. The specimens were transported to the laboratory and stored in 2.5% (w/v) potassium dichromate solution at 4 °C before DNA extraction.

### DNA extraction and PCR amplification

Approximately 200 mg of each fecal specimen was washed at least three times with distilled water by centrifugation at 5000× *g* for 5 min to remove the potassium dichromate. DNA was extracted using the E.Z.N.A.R® Stool DNA Kit (Omega Biotek Inc., Norcross, GA, USA) according to the manufacturer’s instructions. For *E. bieneusi* screening, nested PCR assays were used to amplify an rRNA gene fragment containing the entire internal transcriber spacer (ITS) [6]. Each specimen was analyzed in duplicate using positive and negative controls. The secondary PCR products were examined by electrophoresis in a 1.5% agarose gel and visualized after staining with GelRed™ (Biotium Inc., Hayward, CA, USA).

### Sequencing and phylogenetic analysis

The positive secondary PCR amplicons were sent to a commercial company (GENEWIZ, Suzhou, China) for sequencing. The sequence accuracy was confirmed with bidirectional sequencing, and the sequences obtained were aligned with reference sequences downloaded from GenBank to determine the genotypes, using the program ClustalX 2.0 (http://www.clustal.org/).

The genotypes of *E. bieneusi* isolated in this study were compared with known *E. bieneusi* ITS genotypes with a neighbor-joining analysis in the Mega 5 program [6]. A bootstrap analysis was used to assess the robustness of the clusters using 1000 replicates. The established nomenclature system was used in naming the *E. bieneusi* ITS genotypes [11].

### Nucleotide sequence accession numbers

The nucleotide sequences reported in this paper have been submitted to the GenBank database at the National Center for Biotechnology Information under the accession numbers: MG602791-MG602796.

### Statistical analysis

Chi-square test was used to compare the prevalence of *E. bieneusi* infections and predominant genotypes distributions. Differences were considered significant at *P* < 0.05.

## Results and discussion

Of all 407 individual Bactrian camel fecal specimens, 30.0% (122) were *E. bieneusi*-positive based on the ITS sequence analysis. The majority of the grazing Bactrian camel groups, 14 out of 18 (77.8%), were positive for *E. bieneusi*. Among them, Urumqi-1 had the highest infection rate (84.6%, 11/13) (*χ*^2^ = 67.728, *df* = 17, *P* < 0.001); the other infection rates ranged from 16.7–52.9% (Table 1).

To the best of our knowledge, this is the first report of *E. bieneusi* in Bactrian camels, and the pathogen is widespread in Xinjiang, northwestern China. In China, the average prevalence of *E. bieneusi* in animals ranges from 0.9% (4/426) in rabbits [12] to 45.6% (426/934) in pigs [13]. However, *E. bieneusi* infection has only been reported in some animals in northwestern China (Table 2), the average prevalence ranging from 1.1% (4/353) in white yaks [14] to 47.8% (22/46) in sheep [2]. In Xinjiang, only dairy calves [15] and grazing horses [16] have been previously reported to have *E. bieneusi* infections, with a prevalence of 16.5% (85/514) and 30.9% (81/262), respectively. The high prevalence in Bactrian camels found in this study may be the result of free feeding and drinking water, and mixed feeding with cattle, sheep, goats and other animals in the same pastures, and with the poor veterinary service.

**Table 2:** *Enterocytozoon bieneusi* infections and genotype distributions in animals in northwestern China: summary of previous literature

Region	Host	No. of specimens	No. infected (%)	Genotype (no.)	Reference
Xinjiang	Dairy cattle	514	85 (16.5)	J (57), I (19), BEB4 (4), D (2), EbpC (2), CC4 (1)	[15]
Xinjiang	Grazing horses	262	81 (30.9)	EbpC (21), EpbA (20), BEB6 (9), CHG19 (2), CM6 (4), CM7 (2), CM8 (1), CS-1 (1), CS-4 (1), D (1), G (3), horse1 (4), horse2 (2), O (4), Peru8 (1), XJH1 (2), XJH2 (1), XJH3 (1), XJH4 (1)	[16]
Gansu	White yaks	353	4 (1.1)	BEB4 (2), I (1), WCY1 (1)	[14]
Ningxia	Dairy cattle	109	51 (46.8)	J (25), CM8 (14), I (8), O (1), CHC1 (1), CHC2 (1), CHC3 (1)	[6]
Qinghai	Yaks	327	23 (7.0)	BEB4 (16), I (1), J (1), CHN11 (4), CHN12 (1)	[18]
Shaanxi	Sheep	46	22 (47.8)	BEB6 (4), CHG1 (3), CHG3 (3), CD6 (3), CHG5 (2), E (1), F (1), CHG14 (1), CHG16 (1), CHG24 (1)	[2]
Shaanxi	Monkeys	197	25 (12.7)	D (10), BEB6 (4), MH (7), XH (2), BSH (2)	[19]
Shaanxi	Dairy cattle	198	39 (19.7)	I (21), J (16), CHN1 (1), CSX1 (1)	[20]
Shaanxi	Beef cattle	173	34 (19.7)	I (19), J (14), CSX2 (1)	[20]
Shaanxi	Golden takins	191	28 (14.7)	BEB6 (10), D (8), I (6), TEB1–4 (each 1)	[21]
Shaanxi	Cashmere goats	315	50 (15.9)	SX1 (43), CHG1 (7)	[22]
Shaanxi	Dairy goats	170	56 (32.9)	CHG1 (35), SX1 (13), BEB6 (6), CHG2 (2)	[22]

A total of 14 distinct *E. bieneusi* ITS genotypes were obtained from 122 positive specimens from Bactrian camels. Among them, eight were known genotypes (EbpC, EbpA, Henan-IV, BEB6, CM8, CHG16, O and WL17), and six were novel genotypes (named CAM1 to CAM6). The sequences of the novel genotypes, CAM1 to CAM6, consisted of insertions, deletions, and substitutions compared with known genotypes. In the present study, the novel genotype CAM1 was the most prevalent (59.0%, 72/122) and was significantly predominant (*χ*^2^ = 589.836, *df* = 13, *P* < 0.001) in Bactrian camels in Xinjiang. The other novel genotypes CAM2 (*n* = 8), CAM4 (*n* = 5), CAM3 (*n* = 1), CAM5 (*n* = 1) and CAM6 (*n* = 1) were also identified. The genotype EbpC was the predominant identified of the known genotypes (18.9%, 23/122) in Bactrian camels in Xinjiang, followed by genotype EbpA (*n* = 5). However, the other known genotypes Henan-IV, BEB6, CM8, CHG16, O and WL17, were identified in only one specimen each, though they have been commonly reported in many other types of animals.

**Fig. 1:**
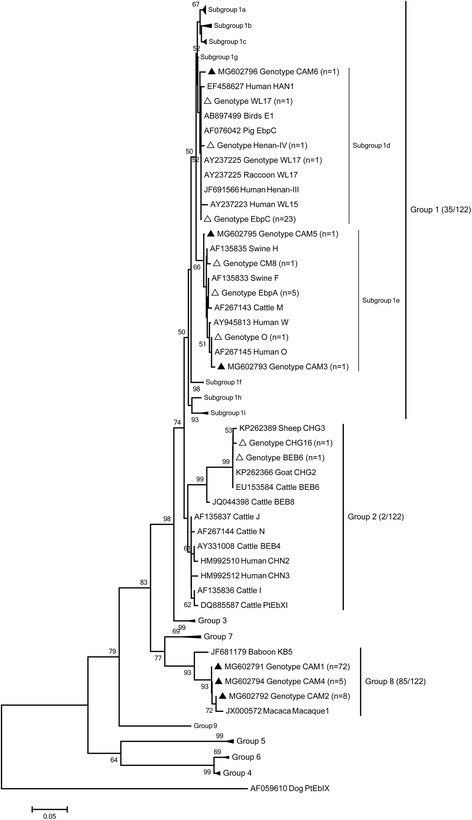
Phylogenetic relationships of the *E. bieneusi* genotypes identified in this study and other reported genotypes. The phylogeny was inferred with a neighbor-joining analysis of the internal transcribed spacer (ITS) sequences based on distances calculated with the Kimura two-parameter model. Bootstrap values > 50% from 1000 replicates are shown at the nodes. The genotypes detected in this study are shown with triangles; previously known genotypes observed in this study are marked with open triangles and the new genotypes are indicated by filled triangles

The phylogenetic analysis of the ITS genotypes revealed the following clusters: Group 1, Group 2 and Group 8. The six known genotypes (EbpC, EbpA, WL17, Henan-IV, CM8 and O) and three novel genotypes (CAM3, CAM5 and CAM6) identified in this study fell into the human-pathogenic Group 1 (Fig. 1), which is the genotype of major zoonotic potential suggesting that the Bactrian camels play a potential role in *E. bieneusi* transmission to humans [11]. In contrast, the two known genotypes CHG16 and BEB6 fell into the cattle host-specific Group 2. The three novel genotypes CAM1, CAM 2 and CAM4 clustered into Group 8 (69.7%, 85/122) (Fig. 1), suggesting that the host-specific genotype CAM1 in Bactrian camels exhibits less zoonotic potential compared to the genotypes clustered into the human-pathogenic group.

In previous studies, *E. bieneusi* genotype EbpC and EbpA were reported in humans and various animals and were also the predominant genotypes in the reports of humans and pigs in China [13, 17]. Genotypes EbpC and EbpA were the most common *E. bieneusi* genotypes in grazing horses in Xinjiang [16], and genotype EbpC was also identified in dairy calves in Xinjiang [15]. Similarly, the *E. bieneusi* zoonotic genotypes EbpC and EbpA were identified in Bactrian camels in the present study. However, there were no published reports of genotypes EbpC and EbpA in animals in Gansu, Ningxia, Qinghai and Shaanxi, northwestern China (Table 2). Further investigations of the epidemiology and host specificity of *E. bieneusi* in humans and other animals in Xinjiang might be informative.

## Conclusions

The present study demonstrated a widespread occurrence of *E. bieneusi* in Bactrian camels in Xinjiang, China. The host-specific genotype, CAM1, was the most predominant genotype, which plays a negligible role in the zoonotic transmission of *E. bieneusi*. The second-most predominant genotype, EbpC, in addition to other genotypes of zoonotic potential, was also commonly identified in Bactrian camels in this study. Bactrian camels could serve as a vector for *E. bieneusi* transmission to humans and other animals, and *vice versa*.

## Abbreviations

ITS: Ribosomal internal transcribed spacer
PCR: Polymerase chain reaction

## References

[1] Keeling P (2009). Five questions about microsporidia. PLoS Pathog.

[2] Shi K, Li M, Wang X, Li J, Karim MR, Wang R (2016). Molecular survey of *Enterocytozoon bieneusi* in sheep and goats in China. Parasit Vectors.

[3] Liu H, Jiang Z, Yuan Z, Yin J, Wang Z, Yu B, et al. Infection by and genotype characteristics of *Enterocytozoon bieneusi* in HIV/AIDS patients from Guangxi Zhuang Autonomous Region, China. BMC Infect Dis. 2017;17(1):684. 10.1186/s12879-017-2787-9 PMC5640944 29029610

[4] Santín M, Fayer R (2011). Microsporidiosis: *Enterocytozoon bieneusi* in domesticated and wild animals. Res Vet Sci.

[5] Guo Y, Alderisio KA, Yang W, Cama V, Feng Y, Xiao L (2014). Host specificity and source of *Enterocytozoon bieneusi* genotypes in a drinking source watershed. Appl Environ Microbiol.

[6] Li J, Luo N, Wang C, Qi M, Cao J, Cui Z (2016). Occurrence, molecular characterization and predominant genotypes of *Enterocytozoon bieneusi* in dairy cattle in Henan and Ningxia, China. Parasit Vectors.

[7] Karim MR, Dong H, Li T, Yu F, Li D, Zhang L (2015). Predomination and new genotypes of *Enterocytozoon bieneusi* in captive nonhuman primates in zoos in China: high genetic diversity and zoonotic significance. PLoS One.

[8] China National Commission of Animal Genetic Resources (2011). Animal genetic resources in China: horses, donkeys, camels.

[9] Liu X, Zhou X, Zhong Z, Deng J, Chen W, Cao S (2014). Multilocus genotype and subtype analysis of *Cryptosporidium andersoni* derived from a Bactrian camel (*Camelus bactrianus*) in China. Parasitol Res.

[10] Sazmand A, Joachim A (2017). Parasitic diseases of camels in Iran (1931–2017) - a literature review. Parasite.

[11] Thellier M, Breton J (2008). *Enterocytozoon bieneusi* in human and animals, focus on laboratory identification and molecular epidemiology. Parasite.

[12] Zhang XX, Jing J, Cai YN, Wang CF, Peng X, Yang GL, et al. Molecular characterization of *Enterocytozoon bieneusi* in domestic rabbits (*Oryctolagus cuniculus*) in northeastern China. Korean J Parasitol. 2017;54(1):81–5. 10.3347/kjp.2016.54.1.81 PMC4792315 26951984

[13] Li W, Diao R, Yang J, Xiao L, Lu Y, Li Y (2014). High diversity of human-pathogenic *Enterocytozoon bieneusi* genotypes in swine in northeast China. Parasitol Res.

[14] Ma JG, Zhang NZ, Hou JL, Zou Y, Hu GX, Zhu XQ (2017). Detection of *Enterocytozoon bieneusi* in White Yaks in Gansu Province, China. Biomed Res Int.

[15] Qi M, Jing B, Jian FC, Wang RJ, Zhang SM, Wang HY (2017). Dominance of *Enterocytozoon bieneusi* genotype J in dairy calves in Xinjiang, northwest China. Parasitol Int.

[16] Qi M, Wang RJ, Wang HY, Jian FC, Li JQ, Zhao JF (2016). *Enterocytozoon bieneusi* genotypes in grazing horses in China and their zoonotic transmission potential. J Eukaryot Microbiol.

[17] Wang L, Zhang HW, Zhao XD, Zhang LX, Zhang GQ, Guo MJ (2013). Zoonotic *Cryptosporidium* species and *Enterocytozoon bieneusi* genotypes in HIV-positive patients on antiretroviral therapy. J Clin Microbiol.

[18] Ma J, Cai J, Ma J, Feng Y, Xiao L (2015). *Enterocytozoon bieneusi* genotypes in yaks (*Bos grunniens*) and their public health potential. J Eukaryot Microbiol.

[19] Du SZ, Zhao GH, Shao JF, Fang YQ, Tian GR, Zhang LX (2015). *Cryptosporidium* spp., *Giardia intestinalis*, and *Enterocytozoon bieneusi* in captive non-human primates in Qinling Mountains. Korean J Parasitol.

[20] Wang X, Wang R, Ren G, Yu Z, Zhang L, Zhang S (2016). Multilocus genotyping of *Giardia duodenalis* and *Enterocytozoon bieneusi* in dairy and native beef (Qinchuan) calves in Shaanxi Province, northwestern China. Parasitol Res.

[21] Zhao GH, Du SZ, Wang HB, Hu XF, Deng MJ, Yu SK (2015). First report of zoonotic *Cryptosporidium* spp., *Giardia intestinalis* and *Enterocytozoon bieneusi* in golden takins (*Budorcas taxicolor bedfordi*). Infect Genet Evol.

[22] Peng XQ, Tian GR, Ren GJ, Yu ZQ, Lok JB, Zhang LX (2016). Infection rate of *Giardia duodenalis*, *Cryptosporidium* spp. and *Enterocytozoon bieneusi* in cashmere, dairy and meat goats in China. Infect Genet Evol.

